# Supplementary material for: Development of a Novel Anti−CD44 Monoclonal Antibody for Multiple Applications against Esophageal Squamous Cell Carcinomas
Source: Int J Mol Sci. 2022 May 16;23(10):5535. doi: 10.3390/ijms23105535 (PMC9146722; doi:10.3390/ijms23105535)
Supplement: Supplementary file 1 [file ijms-23-05535-s001.zip › ijms-1699994-supplementary.pdf]

International Journal of
Molecular Sciences

MDPI

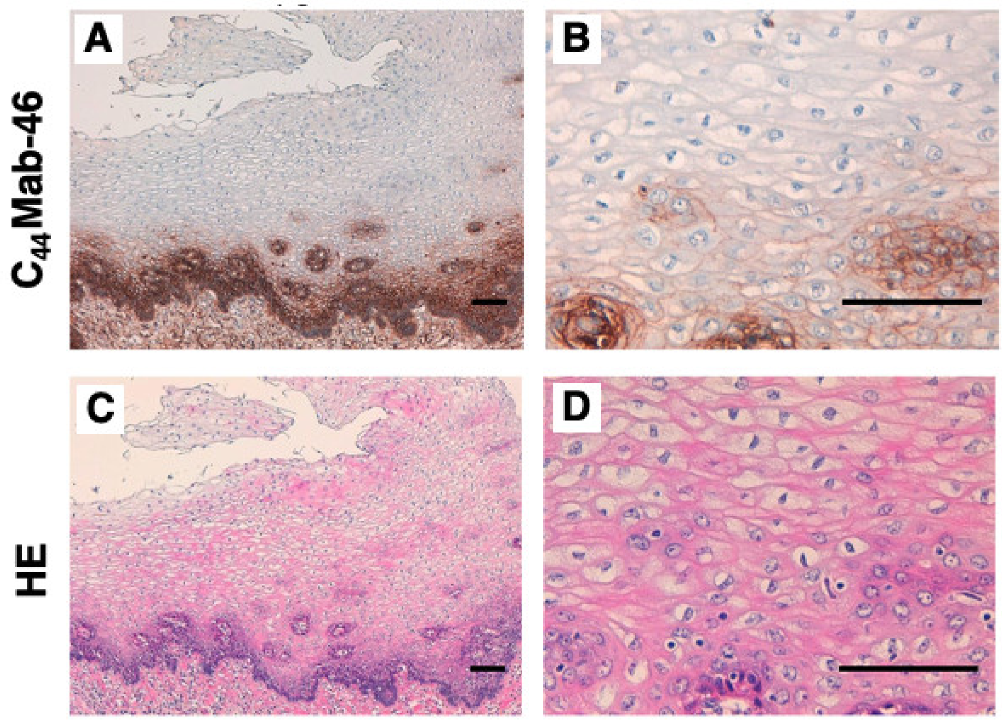

Supplementary Figure S1. Immunohistochemical analysis using C44Mab−46 against normal squamous epithelium of esophagus. (**A**,**B**) After antigen retrieval, sections were incubated with 5 μg/mL of C44Mab−46 followed by treatment with the Envision+ kit. Color was developed using DAB, and sections were counterstained with hematoxylin. (C, D) Hematoxylin and eosin (HE) staining. Scale bar = 100 μm.

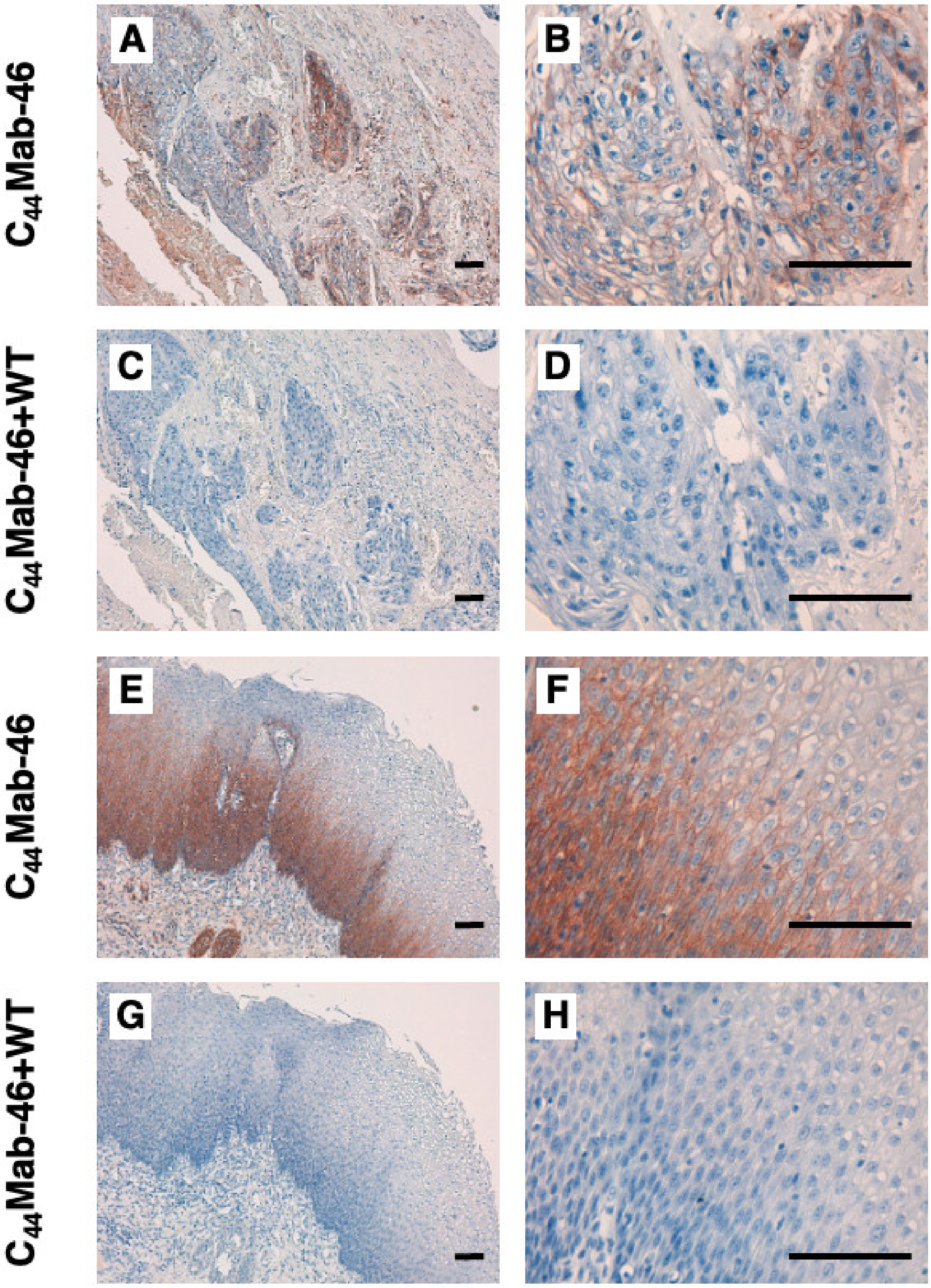

Supplementary Figure S2. Blocking of the C_{44}Mab−46 reactivity to ESCC tissues by the CD44 peptide. After antigen retrieval, sections were incubated with C_{44}Mab−46 (1 μg/ml) or C_{44}Mab−46 (1 μg/ml) plus human CD44 peptide (161-180 amino acids, WT) followed by treatment with the Envision+ kit. Color was developed using DAB, and sections were counterstained with hematoxylin. Scale bar = 100 μm.
